# Focus on oliguria during renal replacement therapy

**DOI:** 10.1007/s00540-024-03342-4

**Published:** 2024-05-22

**Authors:** Qian Zhang, Xiaoting Wang, Yangong Chao, Lixia Liu

**Affiliations:** 1 Department of Intensive Care Unit (ICU), The Affiliated Hospital of Guizhou Medical University, Guiyang, Guizhou 550004 People’s Republic of China; 2 Department of Intensive Care Unit (ICU), Peking Union Medical College Hospital, Beijing, 100005 People’s Republic of China; 3 Department of Intensive Care Unit (ICU), The First Affiliated Hospital of Tsinghua University, Beijing, 100016 People’s Republic of China; 4 Department of Intensive Care Unit (ICU), The Fourth Hospital of Hebei Medical University, Shijiazhuang, Hebei 050011 People’s Republic of China

**Keywords:** Oliguria, Renal replacement therapy, Renal oxygen delivery, Renal oxygen consumption, Acute kidney injury

## Abstract

Oliguria is a clinical symptom characterized by decreased urine output, which can occur at any stage of acute kidney injury and also during renal replacement therapy. In some cases, oliguria may resolve with adjustment of blood purification dose or fluid management, while in others, it may suggest a need for further evaluation and intervention. It is important to determine the underlying cause of oliguria during renal replacement therapy and to develop an appropriate treatment plan. This review looks into the mechanisms of urine production to investigate the mechanism of oliguria during renal replacement therapy from two aspects: diminished glomerular filtration rate and tubular abnormalities. The above conditions all implying a renal oxygen supply–demand imbalance, which is the signal of worsening kidney injury. It also proposes a viable clinical pathway for the treatment and management of patients with acute kidney injury receiving renal replacement therapy.

## Introduction

Acute kidney injury (AKI) is typically defined by either increases in serum creatinine or decreases in urine output. Urine output is often used as a maker of residual renal function in clinical practice, and preservation of residual renal function has been shown to significantly contribute to the health of patients on renal replacement therapy (RRT) [1]. Nonoliguric AKI seems to be associated with better prognosis than oliguric AKI [2]. However, it is observed that AKI patients after starting RRT experienced a further decrease in urine output. The phenomenon has been discussed in the critically ill children literature [3, 4], which has indicated that the duration of RRT was observably longer in patients presenting with oliguria than in non-oliguric patients [3]. Oliguria after onset of RRT is also common in adult patients, just as a post hoc analysis of the ATN Study have shown [5]. And yet urine output is the most commonly described and robust predictor for successful CRRT liberation [6, 7]. Patients who require prolonged RRT have a higher risk of new-onset chronic kidney disease or progression of pre-existing chronic kidney disease, morbidity and mortality from cardiovascular diseases, infection, and end-stage renal disease [8]. In terms of the physiological mechanism for urine production, oliguria is the consequence of diminished glomerular filtration rate (GFR) and increased tubule reabsorption. But under pathological condition, what exactly does a decrease in urine output during RRT mean for the injured kidney?

The review aims to explore the feasible mechanism and clinical impact of lessened urine output after RRT initiating from the perspectives of renal oxygen delivery(DO_2_) and consumption(VO_2_), and to develop a management pathway for oliguria patients.

### Urine formation

Kidney plays a vital role in maintaining metabolic homeostasis. Its functions include excreting waste products, maintaining normal concentrations of electrolytes and water, regulating acid–base balance, and producing hormones and vitamins. Urine formation is a byproduct of kidney function, and also reflects the state of kidney function. In the glomerulus, urine formation begins with the filtration of blood plasma across the capillary walls. The capillary walls permit the filtration of large amounts of fluid and small solutes while preventing the passage of large proteins and blood cells. The overall function of the glomerulus system filters approximately 180 L of fluid a day from renal blood flow. The filtrate is collected by Bowman capsule and delivered to the tubules. And then, most of the filtrate (99% of filtered NaCl and fluid) is reabsorbed by the tubular epithelia [9].

## Characteristics of renal oxygenation and oxygen consumption

### Renal oxygenation

Oxygen tension within organs is influenced by two factors: metabolic demand and oxygen delivery. There are unique features of renal oxygenation that render the kidney susceptible to oxygen demand–supply mismatch and hypoxia. Although the kidneys receive 20–25% of the cardiac output, which is three times higher than myocardial blood flow, renal oxygen extraction ratio is very low (10% of DO_2_) [10, 11], and the vast majority of DO_2_ is used to maintain the kidney’s function (see later section for details)..To preserve osmotic gradients and to enhance urinary, concentration blood flow in the outer medulla is less than 50% of the cortical blood flow in which the former is relatively hypoxic (20 mmHg of the oxygen tension) and the latter relatively hyperoxic (70 mmHg of the oxygen tension) [12]. Thus, under normal physiological conditions, the medulla lives “on the edge of hypoxia”.

### Renal oxygen consumption

As a typical energy-consuming organ, the kidneys require tremendous energy during the process of reabsorption. 70%-80% of VO_2_ is used to support active tubular transport of particularly sodium [13], mainly along the proximal tubule and thick ascending limb, accounting for 60–70% and 25–30% of renal sodium reabsorption, respectively [14, 15]. Tubule transport processes are highly load-dependent. A close linear correlation between GFR, renal sodium reabsorption and VO_2_ has been repeatedly demonstrated [11, 16–18]. Therefore, sodium load after filtration is a crucial determinant of VO_2_. Decreasing GFR and tubular sodium load can decrease tubular sodium reabsorption and VO_2_, and vice versa [19]. Furosemide and atrial natriuretic peptide (ANP), although both induce diuresis, have different effects on VO_2_ due to different pathways. Furosemide reduces sodium reabsorption by inhibiting Na^+^-K^+^-2Cl^−^ cotransporter that is expressed on the apical membrane of the thick ascending limbs of the loops of Henle, resulting in decreases in GFR (-12%), tubular sodium reabsorption (-28%) and VO_2_ (-23%) [16, 20]. ANP ostensibly produces a diuretic effect by inhibiting tubular sodium reabsorption in the collecting duct [21]. However, ANP, by inhibiting aldosterone secretion [22] and reducing plasma renin concentration [23, 24], causes preglomerular renal vasodilation and increases GFR, so it actually leads to an increase in tubular sodium reabsorption (9%) and VO_2_ (26%) [16]. Furthermore, efficiency of sodium reabsorption varies greatly along the nephron. Distal segments are known to require substantially more O_2_ than the proximal tubule to reabsorb the same amount of sodium. The model predicts for the juxtamedullary nephron sodium transport/oxygen consumption ratios of 19.6 for the proximal tubule, 10.5 for the thick ascending limb, 6.5 for the distal convoluted tubule and 3.5 for the connecting tubule [25]. Thus, if proximal tubule transport is compromised, the resulting shift of more sodium to distal sites within the nephron should yield a markedly increase in VO_2_.

**Fig. 1:**
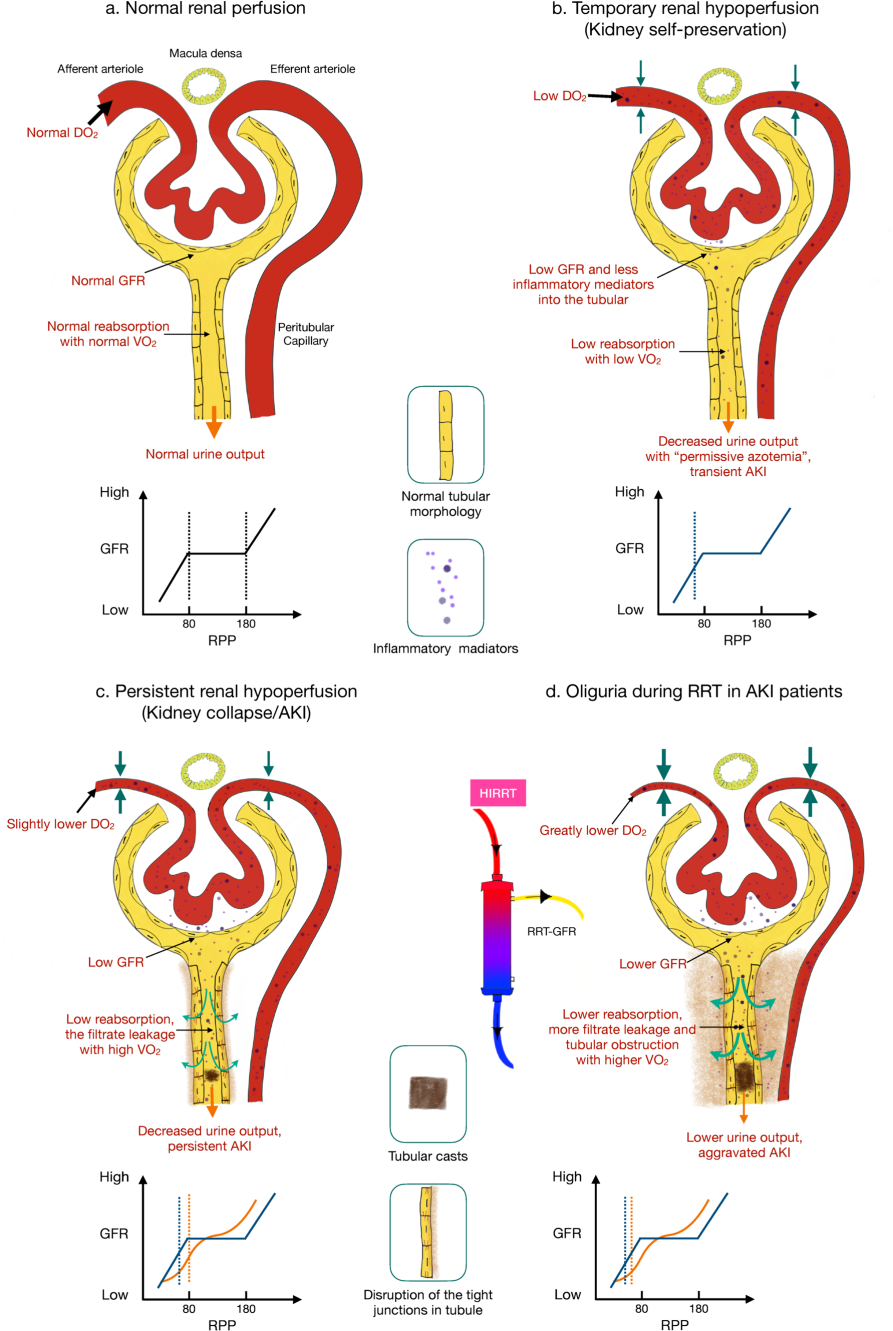
Glomerular filtration and tubular reabsorption in different renal perfusion states. **a** Normal renal perfusion; **b** Transient renal hypoperfusion (renal self-preservation): due to reduction in renal perfusion pressure below the autoregulatory range, constriction of the afferent and efferent arteriole leads to decreased GFR, followed by decreased tubular reabsorption, which is energy conservation. Meanwhile, low GFR reduces the amount of inflammatory mediators into the tubular lumen, which controls the tubular injury. Clinical features include decreased urine output and metabolic waste accumulation in body, which is transient AKI. **c** Persistent renal hypoperfusion: due to persistent reduction in renal perfusion pressure below the autoregulatory range (blue line) or the autoregulatory compromise (yellow line), the afferent and efferent arterioles further constrict, accompanied by a further decrease in DO_2_. As GFR decreases, the amount of solute filtered into the tubule decreases. The tight junctions in the tubule disrupt, leading to filtrate leakage, tubular obstruction and increased VO_2_. These together result in reduced urine output and the persistence of AKI. **d** Oliguria in patients with AKI during RRT: Due to impaired self-regulation, HIRRT causes significant constriction of afferent arteriole, resulting in lower GFR and DO_2_, while further disruption of tight junctions in the tubule leads to more significant filtrate leakage and tubular obstruction, accompanied by an increase in VO_2_ and finally, an sharp decline in urine output. *GFR* glomerular filtration rate, *AKI* acute kidney injury, *DO*_*2*_ renal oxygen delivery, *VO*_*2*_ renal oxygen consumption, *RPP* renal perfusion pressure, *HIRRT* hemodynamic instability during renal replacement therapy, *RRT* renal replacement therapy, *RRT-GFR* glomerular filtration rate produced by renal replacement therapy

On this physiological basis, any risk factor inducing inadequate DO_2_ or increased VO_2_ would harm to the kidneys. Renal autoregulation is critical in maintaining the matching of DO_2_ and VO_2_. The autoregulatory mechanisms maintain renal blood flow (RBF) and GFR independent of renal perfusion pressure over a defined range (80-180 mmHg), which keeps DO_2_ and concentration of Na^+^ entering the tubules stable (Fig. 1a). With the decrease of renal perfusion pressure, the kidneys can exhibit remarkable autoregulation, maintaining constant RBF and consequently GFR to match oxygen supply and demand. However, hypoperfusion occurs when the renal perfusion pressure is below the lower limit of autoregulation, leading to reduced DO_2_, renal tubular ischemia and reduced GFR [26–28].

### Kidney self-preservation and collapse

Hypotension, inflammation and neuroendocrine are the frequent mechanisms affecting renal perfusion [29, 30]. Although RBF appears to be preserved or even increased in sepsis [31, 32], renal microcirculation impairment has also been demonstrated [33]. Watchorn James and colleagues found in patients with septic shock the AKI severity was associated with the degree of renal cortical hypoperfusion, but it did not appear to be associated with alterations in local or systemic blood flow [34]. Interestingly, it has been proposed that a reduced GFR is a way for kidney to protect itself in a low-perfusion state (Fig. 1b). It has been stated that “acute renal success” [35, 36], because reducing the GFR in AKI should lead to a reduction in active tubular sodium reabsorption, which give renal a chance to recover from a negative energy balance and high oxygen demand with a lower risk of further aggravation of ischemia. Furthermore, as highly vascularized organ, the kidneys are placed on the front line to be exposed to systemic inflammatory mediators. The mediators can readily gain access to the tubular space through glomerular filtration [37]. Once into the tubular lumen, the madiators can interact with the tubular epithelial cell through direct or indirect pathways. The epithelium and neighboring tubular endothelial cells are exposed to inflammatory signal to activated, cytokine secreting leukocytes and to other pathogen-associated molecular patterns and damage-associated molecular patterns that ultimately amplify the inflammatory signal and cause greater oxidative stress. Low GFR partially restricts inflammatory mediators into the tubular space and controls the tubular injury. Naturally, low GFR would be accompanied by accumulation of metabolic waste in the body. Recently, the term "Permissive azotemia" has been proposed [38]. Recent evidence suggests that decreased renal clearance does result in significant decreases in monocyte/macrophage infiltration, complement levels, oxidative stress, and other markers of inflammation in the injured kidney [39–41].

Nonetheless, we consider that “acute renal success” can only be used as a temporary emergency plan for the kidney to protect itself facing renal hypoperfusion. Particularly in AKI, the autoregulation capacity is so compromised that the susceptibility to risk factors for hypoperfusion is distinctly increased [42, 43]. In ischemic acute renal failure, the marked change in RBF have been observed over the (renal artery) pressure range of 70 to 125 mmHg [42]. If the risk factors of renal hypoperfusion are not timely removed, the impact of activating tubuloglomerular feedback system (TGF) due to a decrease in sodium ions filtered into the tubules leads to afferent vasoconstriction persistently whereas tubular and interstitial pressure is increased secondary to inflammatory responses, microvascular dysfunction, tubular back-leak, tubular stasis and volume overload. All of these can significantly aggravate the decrease in renal blood perfusion and DO_2_.

Unexpectedly, the decrease in GFR caused by renal hypoperfusion was not accompanied by a corresponding decrease in VO_2_ in AKI. On the contrary, study have shown that VO_2_ is higher for tubular sodium ion reabsorption under pathological context [11]. One can only speculate on the mechanism behind the increased O_2_ utilization for sodium transport in patients with AKI. A potential explanation could be dysregulation of renal tubule paracellular transport. Paracellular transport through the tight junction is a general mechanism for transepithelial transport of solutes in epithelia, including the renal tubule. In renal tubule, paracellular transport leverages the excess free energy in electro-osmotic gradients produced by active transcellular transport to drive additional, paracellular reabsorption of Na^+^, Cl^–^, and other solutes in a purely passive manner, not requiring additional energy expenditure [44, 45]. The tight junction is composed of a complex of multiple proteins, of which the claudins are now believed to form the paracellular pores or channels [44, 46–48]. Claudins are the key integral membrane proteins that mediate paracellular transport [49]. 32% to 64% of the proximal tubules Na^+^ reabsorption is passive and paracellular. Distal segments are known to require substantially more O_2_ than the proximal tubule to reabsorb the same amount of Na^+^, which can be attributed to the substantial paracellular transport along the proximal tubules. Paracellular sodium transport maximizes the efficiency of oxygen utilization in the kidney [50]. Claudins expression is dysregulated in many pathologies including hypoxia, inflammation, oxidative stress [45, 51–53]. Sepsis, toxic and ischemia/reperfusion injuries are the most common causes of AKI and result in disassembly of the tight junctions, increased apoptosis, and tubular cell detachment [45, 54–56]. Another explanation for the increased O_2_ costs for sodium reabsorption in AKI may be renal tubular cell mitochondrial dysfunction [57]. During sepsis, high levels of reactive oxygen species and reactive nitrogen species are produced, and these may overwhelm antioxidant capacity with resultant inhibition of, and damage to, the electron transport chain [58, 59]. VO_2_ may be partially redirected away from adenosine triphosphate production (i.e., oxidative phosphorylation) [57].

It’s predictable that AKI patients combined with persistent renal hypoperfusion would inevitably deteriorate oxygen supply–demand mismatch (low DO_2_ and high VO_2_), which further perpetuates AKI (Fig. 1c).

### Oliguria during renal replacement therapy

Renal replacement therapy is frequently used to treat critically ill patients with AKI. As a group of therapeutic techniques for extracorporeal blood purification, RRT can be used for solute and fluid control while awaiting sufficient recovery of kidney function from AKI. However, the technique does not come without its problems. It may prolong the duration of AKI or impede complete recovery of native kidney function [60]. Even so, there is still no conclusion on how to prescribe RRT optimally, including the timing, modality, and intensity of therapy, which remain the focus of interest.

The secondary analysis of the STARRT-AKI trial suggest that an accelerated strategy of RRT initiation conferred a higher risk of 90-day RRT dependence among critically ill patients with AKI who have pre-existing chronic kidney disease [61]. It is plausible that exposure to RRT may contribute to a higher relative risk of disrupted and maladaptive kidney repair to which patients with pre-existing chronic kidney disease are particularly vulnerable.

Two large randomized controlled trials, the ATN Study and the RENAL Study, both showed that more intensive RRT did not have any beneficial effects on renal recovery, nonrenal organ failure or mortality compared with less intensive RRT [62, 63]. In a meta-analysis, Wang Y el at found that patients remained RRT dependent for longer while receiving higher intensity RRT(a prescribed dose of 35–48 mL/kg/h), compared with standard intensity(a prescribed dose of 20–25 mL/kg/h), in the first 28 days of treatment, which appears to delay renal recovery [64]. Not only that, but in terms of fluid removal, emerging evidence showed that high net ultrafiltration (> 1.75 mL/kg/h) rate group compared with moderate (1.01–1.75 mL/kg/h) and low (< 1.01 mL/kg/h) net ultrafiltration rate groups were associated with lower survival and higher dialysis dependence [65]. Moderate net ultrafiltration rates between 1.01 and 1.75 mL/kg/hour appear to be associated with the lowest risk of death [65]. The above evidences seem to point to the artificial kidney with high intensity of solute or fluid removal would aggravate renal injury in critically ill patients with AKI [66]. More intensive therapy is not also now aligned with guideline recommended application of RRT.

Given the prior studies suggesting a potential relationship between lower urine outputs and adverse outcomes, McCausland et al. seek to explore the relationship of more intensive RRT with urine output in the ATN Study. Compared with the less intensive group (21.5 ml/kg/hr), there was a greater reduction in the daily rate of change in urine output in those receiving more intensive RRT (36.2 ml/kg/hr) [5]. More intensive RRT was associated with an increased risk of a decline in urine output by ≥ 50%. Urine output prior to discontinuation of RRT was the most commonly predictor for successful CRRT liberation [6, 7]. Fernández Lafever Sarah N et al. pointed out that the duration of RRT was markedly longer in patients presenting with oliguria than in non-oliguric patients after RRT [4]. Patients who require prolonged RRT may have more morbidity, mortality and resource utilization than patients who liberate successfully [67–69]. Interestingly, in a randomized, double-blind, placebo-controlled trial, Van Der Voort et al. report that giving furosemide after hemofiltration to increase urine output does not provide any benefit on recovery of renal function in terms of creatinine clearance and duration of RRT [70]. Obviously, when AKI patients receiving RRT develop oliguria, simple diuresis does not improve renal recovery. The first thing to do is to see the essence through the phenomena.

### Related mechanisms for oliguria during renal replacement therapy

Here, we hold that oliguria is the consequence of diminished GFR and tubular abnormalities in AKI patients receiving RRT (Fig. 1d).

### Diminished GFR

We analyzed an potential explanation for the association of more intensive RRT with lower urine output is the renal injury that may arise from more hemodynamic instability during renal replacement therapy(HIRRT), which is common with greater intensity of RRT and is an independent predictor of mortality among critically ill patients [71, 72]. A retrospective study of Mayo Clinic found that hypotension within one hour of RRT initiation was associated with 1.54 times increased odds of in-hospital mortality and 1.36 times increased odds of major adverse kidney events at 90 days [71]. In the ATN trial study, there were more hypotensive events in the more intensive of solute control group. More intensive net ultrafiltration rate with a faster rate or larger volume of fluid removal may be associated with increased hemodynamic instability [73]. Hypotension during RRT could result in recurrent renal ischemia, which might delay recovery of renal function [74].

During RRT, changes in blood composition have different effects on hemodynamics. Water is the most commonly removed component of the RRT process. Although excessive ultrafiltration is a key factor for resulting in HIRRT, recent evidence suggests that multiple other RRT-related factors may precipitate HIRRT [75, 76], mainly including rapid plasma osmolality shifts [77, 78], effect of temperature changes with RRT [79], dialyzer bio-incompatibility [80], clearance of beneficial substances (e.g. vasoactive drugs, “good humors” and so on) [81] and transient myocardial stunning [82]. While the basic mechanisms involved are reduced cardiac output (as a result of hypovolemia or pump failure) and decreased peripheral resistance, HIRRT may be a consequence of multiple mechanisms.

RRT is supposed to support the injured kidney. However, there is a precondition- renal hypoperfusion does not occur. HIRRT is a complication of all RRT modalities commonly used in the intensive care unit. Due to the impairment of renal perfusion autoregulation, AKI patients may be particularly vulnerable to ischemic kidney injury when there is a drop in blood pressure [83]. Along comes the essence of minified renal oxygen supply [42], which can be fatal in patients with AKI. Through activation of the sympathetic nervous system, it leads to elevated activity of the renin–angiotensin–aldosterone system, higher levels of circulating vasopressin and activation of the TGF. This ultimately results in reduced renal filtration and decrease in urine [84].

Therefore, during renal replacement therapy, urine output remains a good indicator of renal perfusion. In this regard, if urine output decreases during RRT, we should further evaluate whether there are factors that cause renal hypoperfusion and correct it, rather than taking it for granted.

### Tubular abnormalities

In addition to understanding the importance of GFR maintenance, from the mechanism of urine formation, tubular abnormalities are also major causes of further oliguria in AKI patients. It is worth stressing that the integrity of the tubular structure determines the effectiveness of renal oxygen utilization. Renal oxygen utilization is inefficient despite decreased glomerular filtration rate and filtered load in patients with AKI. The novel finding by Nakano et al. indicated oliguria occurred in septic AKI patients was due to lipopolysaccharide disrupting tight junctions in proximal tubules, resulting in filtrate leakage from the proximal tubular lumen into the interstitium [45]. In addition, leakage of the filtrate causes the tubular flow downstream to stagnate and reduced shear stress. Apical shear stress has been proven to maintain proximal tubular morphology, including tight junction formation [85, 86]. Inflammatory cytokines are trapped in the renal tubular lumen and induce a tubule-tubule network [87]. Altogether, reduced tubular flow, whether due to leakage of the filtrate or decreased GFR, might create a vicious cycle, leading to further loss of tight junctions in the downstream proximal tubules and increased intrarenal interstitial pressure. Besides, the concentration and stasis of the filtrate in tubules is conducive to the formation of tubular casts. Increased intrarenal interstitial pressure and intracapsular pressure, which is attributed to tubular leakage and tubular obstruction from tubular casts, in turn exacerbates renal hypoperfusion and GFR. This is a vicious cycle leading to more and more tight junction disruption and higher and higher renal interstitial pressure, which could greatly reduce the efficiency of oxygen utilization in process of tubular reabsorption so that VO_2_ increases.

**Fig. 2:**
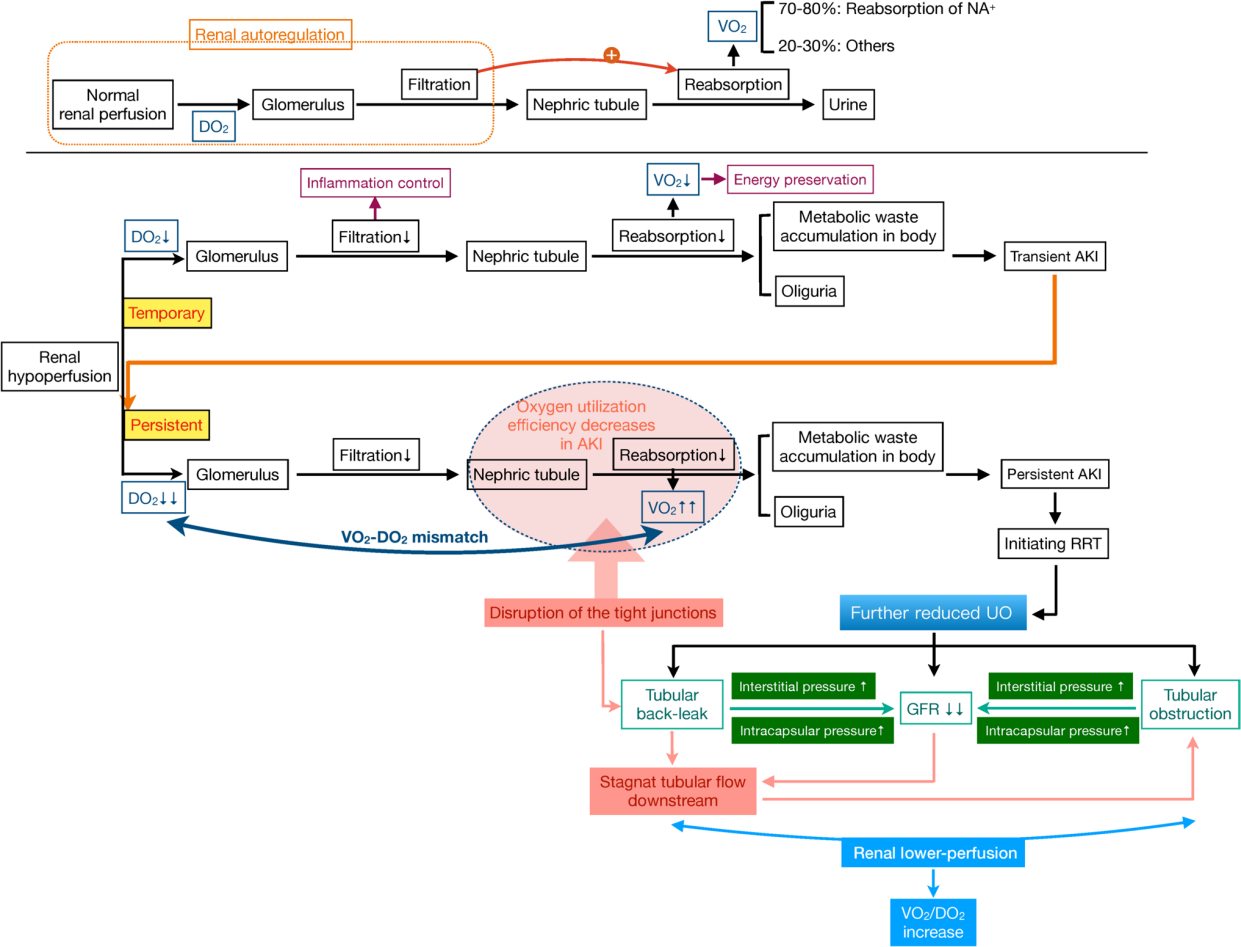
Oliguria during renal replacement therapy signals an imbalance in renal oxygen delivery and consumption. *DO*_*2*_ renal oxygen delivery; *VO*_*2*_ renal oxygen consumption, *AKI* acute kidney injury, *GFR* glomerular filtration rate, *RRT* renal replacement therapy, *UO* urine output

In any case, the decrease of urine output during RRT is a bad signal, implying renal oxygen demand–supply further mismatch in AKI patients, which is predisposing to persistent and worsening renal injury (Fig. 2).

### Management proposals for critical care practitioners

**Fig. 3:**
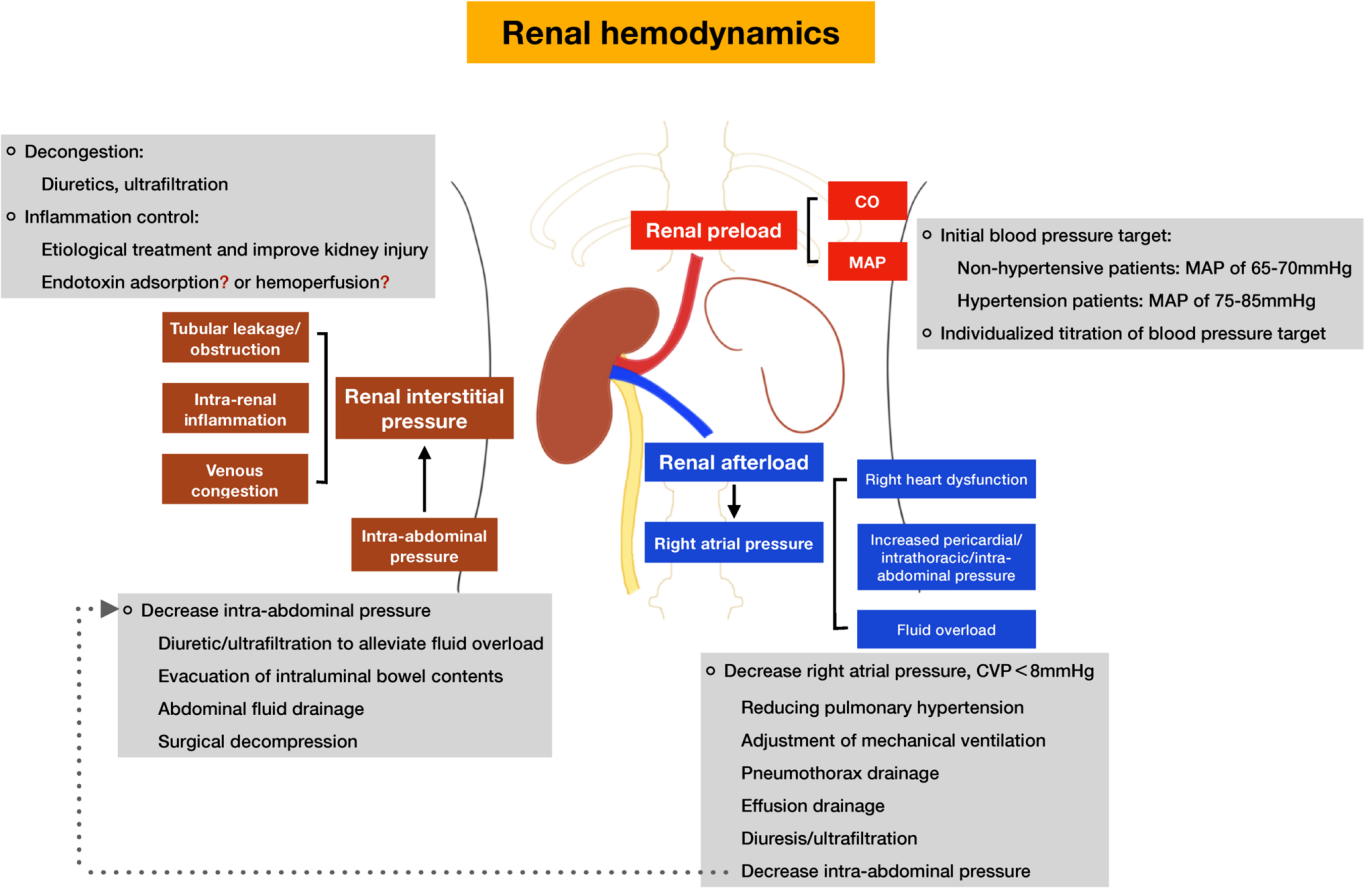
Renal hemodynamics (renal preload, intrarenal pressure and renal afterload) and the therapeutic management of patients with acute kidney injury receiving renal replacement therapy. *CO* cardiac output, *MAP* mean arterial pressure, *CVP* central venous pressure

In the setting of AKI, how to keep the kidneys in a “comfortable” environment in the process of renal replacement therapy will be more conducive to the recovery of the kidneys. To reduce the occurrence of renal hypoperfusion from the perspective of renal hemodynamics, it is recommended to improve the management of AKI patients from the three levels of renal preload, intrarenal pressure and renal afterload, which interact with each other (Fig. 3). During RRT, especially due to more feasible titration management of fluid, artificial kidneys can better assist in achieving the following goals.

### Renal preload

Renal preload refers to blood flow into the kidney, which is the bottom line and the basis of renal oxygen delivery. Organ blood perfusion is mainly determined by both mean arterial pressure (MAP) and cardiac output. As an organ with strong blood flow autoregulation, renal blood perfusion is more susceptible to MAP fluctuations. Kidneys can be effectively perfused when the blood pressure or perfusion pressure is above the lower limit of autoregulation. Poukkanen and colleagues found that an MAP value less than 73 mmHg was associated with the occurrence and progression of AKI in sepsis patients, and almost half of the patients had a history of hypertension [88]. Unless the patients has a history of chronic hypertension, ideally, the target of vasopressor therapy should be a MAP of 65–70 mmHg [89].

### Renal interstitial pressure

A variety of pathological conditions may elevate renal interstitial pressure, such as tubular leakage, tubular obstruction, intra-renal inflammation, and venous congestion [90–92], which has worse GFR and renal outcome and higher mortality. Reduced renal interstitial pressure effectively prevents functional and structural renal impairment [93]. Decongestion is the cornerstone in treatment, such as diuretics and ultrafiltration. Moreover, etiological treatment and control kidney injury are effective ways to relieve intra-renal inflammatory response, while the existing methods of removing inflammatory mediators in the body through blood purification still lack sufficient evidence. Besides, renal interstitial pressure is also affected by extrarenal pressure- intra-abdominal pressure. Increased intra-abdominal pressure results in decreased RBF, GFR, tubular function, and urine output as well as possible difficulties with breath and maintenance of cardiac output [94, 95]. Once intra-abdominal hypertension is recognized, some measures can be undertaken to reduce intra-abdominal pressure (diuretic/ultrafiltration to relieve fluid overload, evacuation of intraluminal bowel contents, drainage of abdominal fluid, even surgical decompression).

### Renal afterload

Renal afterload refers to the resistance of renal venous blood return. The right atrium serves as the endpoint for abdominal organs venous blood return. The elevated right atrial pressure obstructs the return of blood from the renal vein, resulting in renal venous congestion. Studies demonstrated that renal venous congestion showed a relationship with reduction in urine flow and alteration in glomerular and tubular function. Through backward conduction of pressure, increased renal venous pressure leads to renal parenchymal congestion within the non-distensible renal capsule giving rise to increased interstitial pressure that decrease GFR and renal perfusion [96]. Renal venous congestion often is reflected by high central venous pressure (CVP). Elevated CVP is an independent risk factor for the occurrence and progression of AKI. AKI is less frequently in patients with CVP < 8 mmHg [91]. Strategies to reduce CVP include improving right heart function (reducing pulmonary hypertension), reducing pericardial/intrathoracic/intraabdominal pressure (effusion drainage), and diuresis/ultrafiltration to reduce fluid overload.

## Conclusions

Urine output after RRT initiation is influenced by all kinds of factors, particularly the underlying renal status, including the negative effects of various pathologic factors on glomerular filtration rate and tubular structure as well as function, which can hinder renal recovery. From a renal hemodynamic perspective, we propose a set of possible clinical approaches to help better manage these patients.

## Data Availability

Not applicable.

## Abbreviations

RRT: Renal replacement therapy
AKI: Acute kidney injury
RBF: Renal blood flow
DO_2_: Renal oxygen delivery
VO_2_: Renal oxygen consumption
GFR: Glomerular filtration rate
ANP: Atrial natriuretic peptide
TGF: Tubuloglomerular feedback system
HIRRT: Hemodynamic instability during renal replacement therapy
MAP: Mean arterial pressure
CVP: Central venous pressure
